# Viral Escape from a Candidate HIV-1 Vaccine Targeting Protease Cleavage Sites Is Associated with a Dramatic Fitness Loss in SIVmac239-Infected Cynomolgus Macaques

**DOI:** 10.3390/v18030370

**Published:** 2026-03-17

**Authors:** So-Yon Lim, Ma Luo, James B. Whitney

**Affiliations:** 1 Beth Israel Deaconess Medical Center, Harvard Medical School, Boston, MA 02215, USA; 2 Medical Microbiology and Infectious Diseases, University of Manitoba, Winnipeg, MB R3E 0J9, Canada; Ma.Luo@umanitoba.ca

**Keywords:** HIV vaccine, rhesus model, SIVmac251, viral fitness

## Abstract

A novel HIV-1 vaccine candidate under development targeting the highly conserved protease cleavage regions reduced viral acquisition and delayed disease progression in a macaque SIV-challenge model. Breakthrough virus isolated from vaccinees and control animals were sequenced in the regions surrounding the SIV protease cleavages. We identified unique viral mutations that were associated with alterations in viral load and maintenance of CD4+ T cell counts in vaccinees. To evaluate whether the vaccine-elicited mutations were detrimental to virus fitness, we produced 11 mutant constructs and transfection-derived viral stocks harboring mutations in both PCS2 (in CA/p2) and PCS12 (in Nef) that had emerged at high frequency during breakthrough viremia. Virus preparations harboring mutations displayed impaired proteolytic Gag processing, reduced viral RNA incorporation and p27-CA content. These mutants were also compromised in their ability to replicate in primary cells and cell lines. Interestingly, we observed only partial compensation of these PCS2 defects by downstream mutation at PCS12. In sum, we demonstrate that vaccine-elicited immunity directed to viral protease cleavage regions impair viral escape, and breakthrough virus cannot easily restore replicative fitness.

## 1. Introduction

HIV-1 infection is a major public-health concern. According to the statistics updated by the World Health Organization (WHO) and the Joint United Nations Program of HIV/AIDS (UNAIDS) in 2024, an estimated 39.9 million people were living with HIV globally, with 1.3 million new infections and 630,000 AIDS-related deaths, underscoring the ongoing need for sustained global efforts in prevention, treatment, and care. An effective vaccine to prevent HIV-1 infection is still considered key to realizing an end to the epidemic. Although there has been considerable progress in vaccine development, we do not yet have a vaccine modality capable of controlling HIV-1 transmission [1,2,3,4,5,6,7,8]. Despite the challenges in development, the findings of the RV144 trial demonstrated that clinical efficacy of vaccine regimen using a prime-boost strategy combining a recombinant canarypox vector vaccine (ALVAC-HIV) with a recombinant gp120 protein boost (AIDSVAX B/E), albeit limited, was possible [9,10,11,12,13,14,15].

In prior studies of the Pumwani sex worker cohort, natural resistance to HIV-1 infection was observed and it was demonstrated that several human leukocyte antigens (HLAs) and unique HIV-1 specific T-cell responses were associated with this resistance [16,17]. Interestingly, the Gag peptide recognized by the protective HLA allele with relatively high affinity was a 9-mer peptide that spanned the protease cleavage site (PCS) at p17/p24 [16]. We recently conducted a candidate vaccine study, based on observations from the Pumwani sex worker cohort, in a translational SIV-macaque vaccine model. The PCS vaccine moderately reduced viral acquisition in female Mauritian cynomolgus macaques challenged by the intravaginal route with SIVmac251. Vaccine-induced T cell responses were noted as key correlates [18,19,20]. The HIV-1 protease mediates the cleavage of Gag, Gag-Pol and Nef precursor polyproteins in a highly specific, temporarily regulated manner. Gag cleavage reactions are required to morphologically rearrange an immature particle into a mature, infectious virion. For this reason, any mutations resulting in amino acid substitutions in these cleavage sites influence proteolytic processing and impact viral infectivity. However, data on how HIV-1 cleavage site directed-immunity impacts viral fitness are limited [16,20,21].

Our candidate vaccine used in the study is a modified recombinant vesicular stomatitis vector and nanocarriers to deliver twelve 20-amino acid peptides, specifically −10/+10 regions flanking each cleavage site of SIVmac239. The results showed that vaccine-elicited responses targeted the PCS epitope regions and exerted selective immune pressure leading to specific viral mutations, which likely act early in the viral replication cycle. To fully understand the impact of these vaccine-driven mutations on viral fitness, we investigated the relative frequencies of mutations at the amino acid level within Gag, Gag-Pol and Nef and their associated fitness costs on breakthrough virus isolated from vaccinees following SIV challenge [22,23].

## 2. Materials and Methods

**Construction of recombinant provirus.** One or two nonsynonymous nucleotide changes were introduced into the representative PCS of the infectious molecular clone SIVmac239 with the QuikChange XL site-directed mutagenesis kit (Stratagene, Lexington, KY, USA) according to the manufacturer’s instructions. Changes introduced at positions with polymorphisms in SIV sequences were identified by 454 pyrosequencing of viruses isolated from plasma following SIV infection. Ten unique clones were generated and sequenced throughout the viral genome to confirm that the changes were introduced and that the sequences were identical to that of wild-type (WT) SIVmac239 at all other positions. Five of the PCS mutant clones contained single amino acid mutations in either the PCS12 region (-8R or -8E) or PCS2 cleavage site (-8, -7 and -6). The other five PCS mutant clones contained two amino acid mutations, one in PCS2 and the other in PCS12 region.

**Cell culture and transfection and virus stocks.** Individual infectious stocks of PCS mutant variants and WT were generated by transfecting the 293FT cell line (Life Technologies, Carlsbad, CA, USA) with the corresponding full-length viral molecular clone plasmid with the Lipofectamine2000 (Life Technologies, Carlsbad, CA, USA) according to the manufacturer’s instructions. Culture medium was changed at 24 h post-transfection. At 48 h post-transfection, virus-containing supernatant was clarified by centrifugation, sterilely filtered through a 0.45 μm filter, aliquoted, and stored at −80 °C.

**Viral RNA assays and p27 enzyme linked immunosorbent assay (ELISA).** Virus content in culture fluids was monitored by both RT-PCR and SIV p27 antigen capture assay. Viral RNA was isolated from cell-free culture supernatant using a viral RNA extraction kit (Qiagen, Germantown, MD, USA) and was quantitated using RT-PCR in parallel with a SIV-gag RNA standard as described [24]. The SIV p27 antigen in the culture supernatant was quantitated using antigen capture ELISA kit (Zeptometrix, New York, NY, USA) according to the manufacturer’s instructions.

**In vitro infectious titer.** The titers (50% tissue culture infectious dose (TCID_50_) of all stocks were determined in CEMx174 cell (NIH AIDS reagent program) culture and data was analyzed using the method described by Spearman and Karber [25].

**Western blotting of viral protein.** At 48 h post-transfection, virus-containing culture fluids from 293FT-transfected cells were collected and clarified. Virus was then purified by pelleting through 20% sucrose cushion using ultracentrifugation at 110,000× *g* for 1 h at 4 °C in a Beckman ultracentrifuge (Beckman Coulter, Inc., Brea, CA, USA). 293 FT transfected cells were lysed in NP-40 lysis buffer (Boston BioProducts, Milford, CT, USA). Total protein in both virus and cell lysates was measured using a BCA assay kit (Thermo Fisher Scientific, Cincinnati, OH, USA) and equivalent amounts were separated by SDS/PAGE and visualized with monoclonal anti-p27 antibody (NIH clone 55-2 F12 MCH) and HRP-conjugated anti-mouse IgG secondary antibody (Sigma-Aldrich, St. Louis, MO, USA), respectively. Levels of β-actin were also assessed as a control for the loading of total protein in cell lysates by using anti-β-actin antibody (Sigma-Aldrich). Densitometric analysis of Western blotting was performed using NIH image v1.63 software.

**Virus replication in CEMx174 cells and rhesus macaque PBMCs.** Viral stocks were thawed and treated with 100 U of DNase I in the presence of 10 mM MgCl_2_ at 37 °C for 0.5 h to eliminate any residual contaminating plasmid DNA prior to inoculation of cells. Infection of CEMx174 cells was performed by incubating 10^6^ cells with 1 ng of viral p27 antigen equivalent for 2 h at 37 °C. Infected cells were then washed twice with phosphate-buffered saline and re-suspended in fresh supplemented RPMI-1640 medium. Cells were split at a ratio of 1:3 twice per week. Virus replication was also assessed in peripheral blood mononuclear cells (PBMCs) isolated from two rhesus macaques (ID: T633, T638). Briefly, 1 × 10^6^ ConA-activated rhesus macaque PBMCs were infected with SIV stocks containing 3 ng of p27-CA equivalent at 37 °C for 2 h. Cells were then washed extensively to remove remaining virus. Cells were maintained in 2 mL of culture medium and fresh stimulated PBMCs were added to the cultures on day 7 post-infection. Virus production in culture supernatant was monitored by both RT-PCR and SIV p27 antigen capture assay.

**Growth-competition assays.** The assay was performed in 12-well plates seeded with 2 × 10^6^ rhesus T-cell lines that transformed with *H. papio* in 2 mL total volume. Viral competition was conducted essentially as described [26]. The two viruses under evaluation including the reference virus (SIVmac239 Vif_mut_) and each PCS mutant virus were added to the target cells at an individual MOI of 0.002, which is accepted to be low enough to prevent recombination. Each data point was derived from triplicate cultures on the same plate, and all experiments were performed three times. Competition cultures were maintained for 9 days, with supernatants taken on days 1, 2, 3, 5, 7 and 9. Virus production in culture fluids was monitored using q-PCR.

**Monitoring viral mutation by pyrosequencing.** PCR primers were designed to incorporate the Lib-L fusion tag for use with the GS Titanium Lib-L emPCR kit (Roche, Indianapolis, IN, USA) in addition to a specific 10-base multiplex identifier (MID) tag sequence in order to facilitate demultiplexing the sequencing reads after pooling. Each MID tag sequence specifies a macaque’s post-infection time point and was included in each primer pair targeting a specific protease cleavage site (Supplemental Table S2). For example, the primer pair PC-1-F-MID-1 and PC-1-R-MID-1 amplified a product containing protease cleavage site 1 with a MID tag sequence that referred to a macaque’s first post-infection time point. Primer pairs were designed and optimized using Primer-BLAST [27]. Viral RNA was reverse-transcribed and amplified using the Enhancer Avian HS RT-PCR kit (Sigma-Aldrich) with optimizations following the manufacturer’s instructions to amplify 200 to 304 nucleotide sequences around the 12 PCS region. Briefly, 8 µL of viral RNA was reverse-transcribed with random nonamers. Two microliters of first strand cDNA template was amplified using touch down PCR amplification with primer pairs. The touchdown PCR conditions were as follows: 94 °C (2 min); 9 cycles of 94 °C (15 s), 65 °C (30 s) with −1 °C/cycle, 68 °C (30 s); then 24 cycles of 94 °C (15 s), 55 °C (30 s), 68 °C (30 s), followed by 68 °C (5 min). Amplified products were diluted 50-fold, and 2 µL was used as template for second round touchdown amplification in a 50 µL reaction volume with above conditions. PCR products were visualized by agarose gel electrophoresis, purified using the Agencourt AMPure XP system (Beckman Coulter, Brea, CA, USA), quantitated with picogreen and normalized to a standard concentration prior to pooling. Equal molar amounts of all products for a given animal were pooled to form 16 individual pools. These were sequenced on a GS FLX Titanium sequencer (Roche, Indianapolis, IN, USA) with one pool per region on a 16-region run.

**Viral mutation analysis.** The low-quality base and Ns (with quality score < 23) were trimmed off and short reads (<100 bps) were removed before mapping the sequencing reads to the reference sequence of SIVmac239 using Blast. All sequences were edited before mapping the sequencing reads to the SIVmac239 reference using Blast. Only viral sequence mutations (including frameshift mutations) with frequencies more than 1% were used for downstream analysis. Sequence reads with frameshift mutations (excluding the homopolymer region) were identified and only sequence reads without frameshift mutations were used for downstream analysis.

## 3. Results

**Viral cleavage site mutations in vaccinees after SIV challenge.** The PCSs exhibit a high degree of conservation across SIV strains that differ in their pathogenicity and host specificity. Amino acid sequence alignment of residues overlapping cleavage sites from multiple SIV isolates that were frequently used in non-human primate HIV-1 models are shown (Supplemental Figure S1A,B). In the present study, we systematically analyzed the sequences of breakthrough virus from both vaccinated and control cynomolgus macaques following SIVmac239 challenge. We obtained an average of 512 sequence reads per each cleavage site across 200–304 bp segments spanning the −10/+10 amino acids flanking each of twelve PCSs of SIVmac239. Only viral sequence mutations observed in more than two animals were used for downstream analysis. The deep coverage created a high-resolution view of the viral mutation, not only revealing a large number of mutations but also capturing the frequency of each mutation within the population.

**Figure 1:**
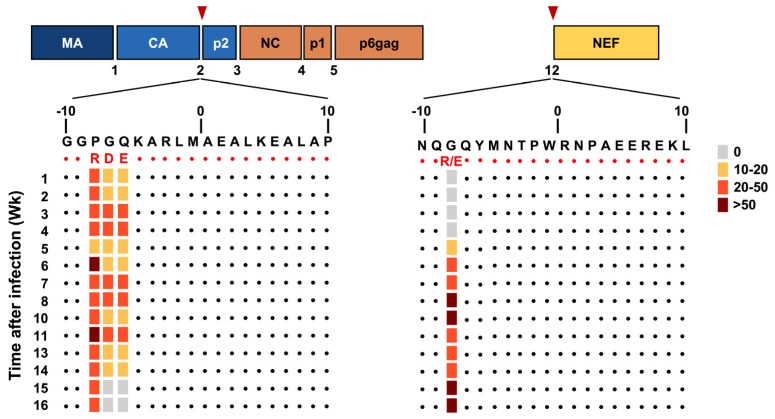
**A schematic illustrating protease cleavage sites in Gag and Nef and representative amino acid sequence changes at each site.** Protease cleavage sites (PCS) in the Gag polypeptide and Nef are indicated. Locations of two major mutation sites corresponding to PCS2 and PCS12 are indicated by inverted triangles. The percentage of SIV with high rates of polymorphism (>30%) in a PCS site isolated from 11 vaccinated animals are indicated. The SIVmac239 reference sequence is shown at the top in black and predicted amino acid (AA) substitutions of nonsynonymous mutations are shown below the reference sequence in red. Each dot indicates a position identical to the reference.

Following repeated, escalating-dose intrarectal challenge with SIVmac239, 100% (11 of 11) of animals infected in the vaccine group harbored SIV populations with a mutation(s) at more than one PCS compared to 60% (3 of 5) of CMs from the control group. We conducted an analysis to compare the PCS derived from the wild-type virus and those isolated from monkeys in both vaccine and control groups of this study. When compared to the control group, viruses isolated from vaccinated monkeys contained a significantly higher rate of non-conservative amino acid substitutions in PCS2 and PCS12 (Supplemental Figure S2). The frequencies of each mutation in each cleavage site varied among monkeys in the vaccine group, demonstrating the extensive heterogeneity of the viral population (Supplemental Figure S3). The major mutation located eight amino acid downstream of the PCS2 site (PCS2(-8)) was present in ≥20% of the total viral population through 16 weeks post-infection, while the mutations located either seven or six amino acids downstream of the PCS2 site (PCS2(-7), PCS2(-6)) were present at low frequency up to 14 weeks post-infection (Figure 1).

**Figure 2:**
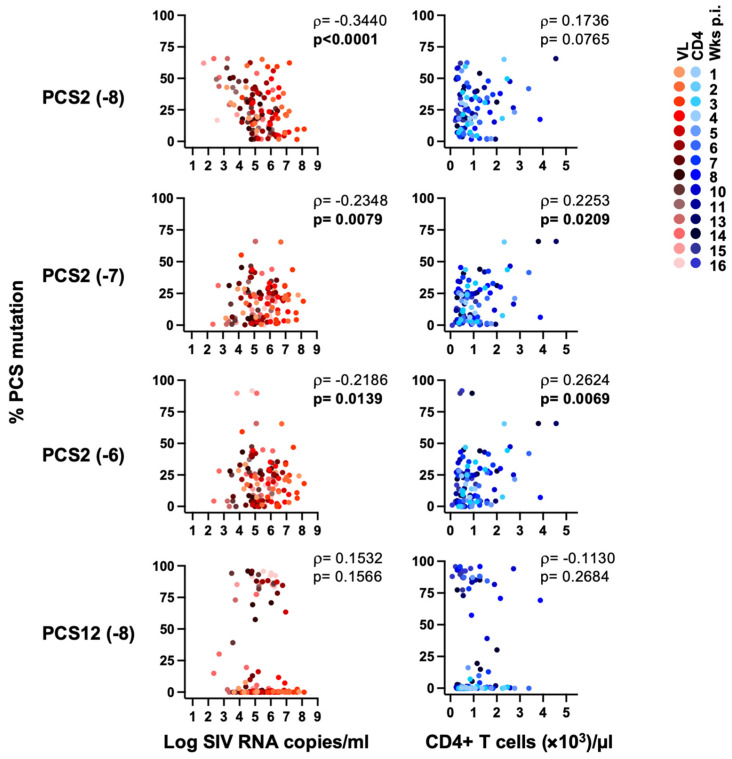
**Association of PCS mutations with plasma SIV RNA and CD4+ T cell count following SIV infection.** Individual relationship between the prevalence of four major amino acid mutations in PCS2 and PCS12 and either plasma SIV RNA copies or CD4+ T cell counts is shown. Correlation plots represent sets of repeated data measures within eleven vaccinees between 1 and 16 weeks post-infection. Spearman correlation was performed and both correlation coefficient (ρ) and *p* value are shown.

Animals in the vaccine group exhibited 10–50% mutation rates in the two latter positions. Interestingly, mutations at the site cleaving Nef (PCS12) emerged as early as 5 weeks after SIV infection and were selected at high frequencies (≥20%) through 16 weeks post-infection. All mutations at both PCS 2 and PCS12 were selected in vaccinated animals rather than in the controls. The changes in the relative frequencies of vial variants following SIV challenge may reflect the fitness costs of specific mutations or selective pressures exerted by the host in the vaccine group. To investigate this, the frequency of sequence reads containing mutations and amino acid substitutions were correlated with either viral load or CD4+ T cell count (Figure 2). Three unique mutations in PCS2 (-8, -7 and -6), but not in PCS12, were associated with reduced viral load and maintenance of CD4+ T cells in the vaccinees [20].

**Generation of PCS mutant clones and in vitro characterization of transfection-derived PCS mutant virus.** We constructed a series of mutations within two cleavage sites, PCS2 in CA/p2 and PCS12 in Nef and then cloned them into the wild-type (WT) SIVmac239 backbone. We then produced eleven transfection-derived viral stocks comprising each of the 10 PCS mutants and WT virus as a comparator (Supplemental Table S1). Two of these PCS mutant stocks contained single amino acid mutations in PCS12 region (-8R or -8E), which cleaves Nef. Three mutant stocks contain single amino acid mutation in PCS2 (-8R, -7D and -6E). The remaining five PCS mutant stocks contained two amino acid mutations, one in PCS2 and the other in PCS12 region. SIV-CA ELISA and RT-PCR were used to evaluate each of these full-length mutant viral stocks for viral p27-CA content and SIV RNA levels, respectively.

**Figure 3:**
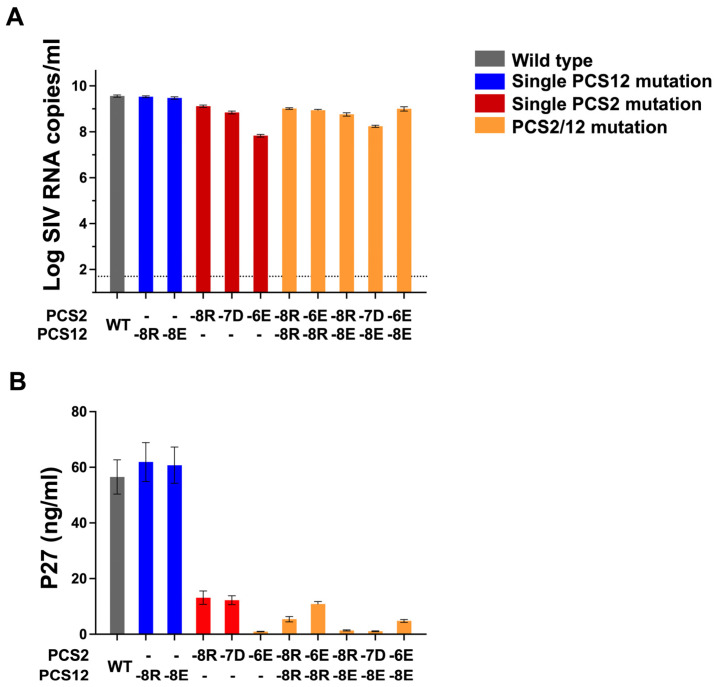
**RNA and protein content between PCS mutant and WT virus.** Eleven transfection-derived viral stocks comprising the 10 PCS mutant clones that were generated and WT SIVmac239 as a comparator were tested for viral contents. Five of these PCS mutant stocks contain single amino acid mutation in either PCS12 region (-8R or -8E) or PCS2 cleavage site (-8, -7 and -6). The other five PCS mutant stocks contain two amino acid mutations, one in PCS2 and the other in PCS12 region. Results of RT-PCR and SIV-CA ELISA are shown for SIV RNA level (**A**) and viral p27-CA (**B**) content of each full-length mutant viral stock. Error bars represent the standard deviation of two separate measurements. The values of viral RNA and p27 from the groups of virus mutants were compared to those of WT SIVmac239.

Next, we compared the viral RNA and p27 CA protein content in the five PCS mutants harboring a single amino acid change and compared to WT SIV expression levels. The two PCS 12 mutant stocks contained 3.40 × 10^9^ and 3.32 × 10^9^ RNA copies/mL and 62 and 61 ng p27-CA/mL, respectively. No significant differences in these two measurements were observed between clones harboring single PCS12 mutations and the WT condition. However, compared to WT, all three single PCS2 mutant virus preparations contained significantly lower viral RNA, ranging between 6.83 × 10^7^ and 1.31 × 10^9^ RNA copies/mL (Figure 3A). Moreover, we found that each (of 3) PCS2 mutants contained significantly reduced p27-CA content ranging from 0.9 to 13 ng/mL, implying a significant impairment to SIV-CA protein production in these variants (Figure 3B). We next evaluated the viral RNA and p27-CA protein content in the remaining five PCS mutant stocks harboring combinations of two separate amino acid mutations, one introduced into the PCS2 site and the other within the PCS12 region. The observed reduction in viral RNA content or p27-CA values between WT and each double mutant were similar to observations between single PCS2 mutant virus preparations (Figure 3A,B). Overall, we demonstrate only weak partial compensation of the single mutation in PCS2 by the inclusion of the PCS12 mutation.

**Figure 4:**
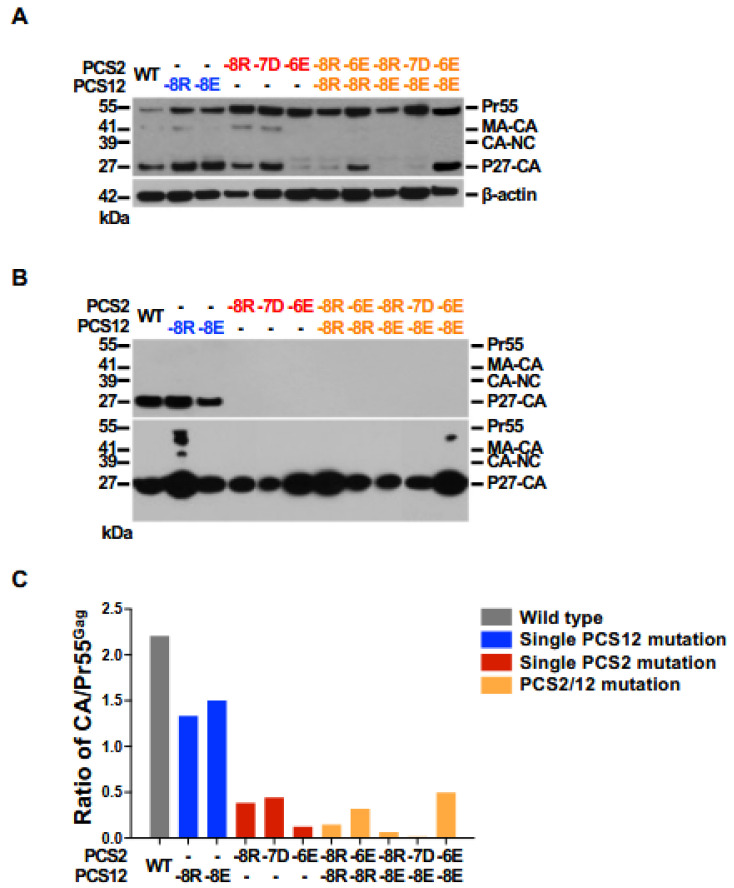
**Proteolytic processing of Pr55^Gag^ of PCS mutant virus.** Virus or cell lysates prepared from 293FT cells transfected with 10 PCS mutants and wild-type clones were resolved by SDS-PAGE and visualized by Western blotting. The processing of Pr55^Gag^ precursor in either cell lysates from transfected 293FT cells (**A**) or pelleted SIV virions, shown in two exposures: upper panel, 10 ng total viral protein/lane; lower panel, concentrated viral protein equivalent to 3 ng of p27/lane (**B**) derived from each viral stock are shown. The detected SIV viral proteins and their corresponding molecular weight are indicated in panels. β-actin was used as a loading control to confirm equal protein loading across lanes. The ratio of p27 CA to Pr55^Gag^ that quantitatively analyzed by densitometer are shown (**C**).

**Gag processing of SIV-carrying mutations in PCS2 (CA/p2).** We evaluated the impact of each amino acid substitution on the activity of the protease to process Gag polyprotein. We performed Western blotting for p27-CA on either the cell lysates of 293FT cells producing mutation-containing molecular SIV clones and on cell-free virus pellet harvested two days after transfection of 293FT cells. A dense p27 signal was detected in the cell lysate from cells transfected with WT SIV. Cell lysates from cells transfected with molecular clones carrying single mutations in PCS2 contained substantially decreased amounts of p27. The two clones carrying a single PCS12 mutation (-8R and -8E) had comparable amounts of p27 (Figure 4A).

Consistent with these findings, a significant amount of p27-CA was detected in pelleted virus from the supernatants of both WT SIV and two PCS12 mutants (Figure 4B, upper). In contrast, supernatants from PCS2 mutants were assessed and p27-CA was not detected when equivalent amounts of total protein (10 ng) were loaded; p27-CA proteins were seen in all of these clones only when substantially more total protein was concentrated (overloaded) to obtain equivalent p27 (3 ng) as detected by ELISA and then loaded (Figure 4B, lower). All clones harboring either a single amino acid mutation in PCS2 or two amino acid mutations, one in PCS2 and the other in PCS12 region, had decreased p27-CA in cell lysates. When analyzed by densitometry, the p27-CA decrease in these mutant viruses ranged between 45 and 87% (Figure 4C).

**Figure 5:**
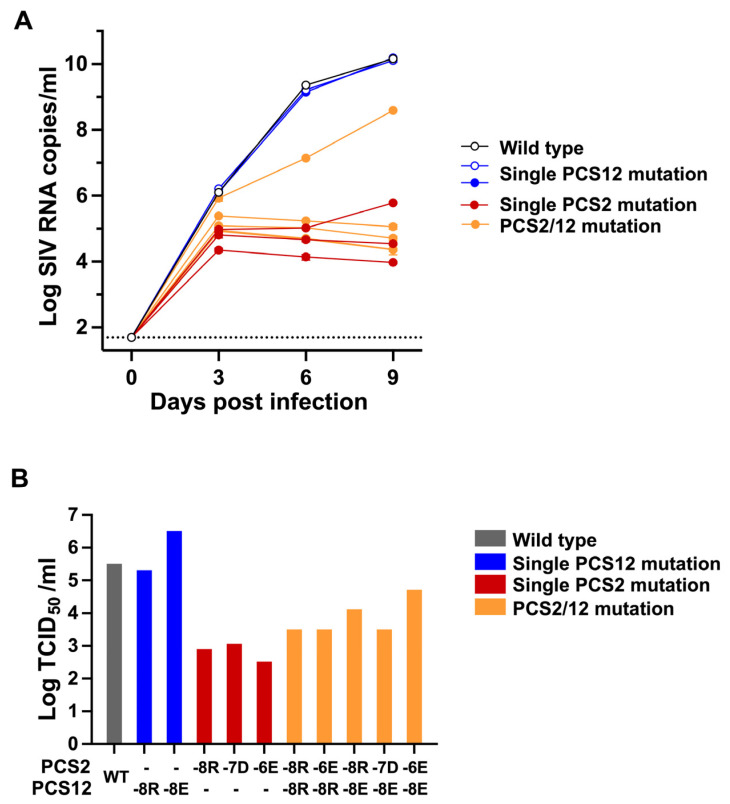
**Viral replication of PCS mutant viruses in CEMx174 cells.** (**A**) Replication of PCS mutant viruses in CEMx174 cells. CEMx174 cells were infected with 1 ng p27 equivalents. Viral replication was longitudinally monitored by RT-PCR for SIV RNA in culture supernatants. (**B**) TCID_50_ analysis of viral infectivity. Replication of virus in CEMx174 cells was assessed by measuring SIV p27 antigen in culture supernatants. TCID_50_ of each virus stock was calculated using the Spearman–Karber method. The horizontal dotted line represents the limit of detection of SIV RNA, which was 50 SIV RNA copies/mL.

**Replication of PCS mutants.** In order to examine the effect of these mutations on viral replication, we assessed the growth kinetics of each infectious clone in CEMx174 cells. As shown in Figure 5A, all molecular clones harboring PCS mutations were capable of replicating in culture, as measured by SIV RNA in culture supernatants. While single PCS12 clones appeared to replicate comparably to WT SIV in CEMx174 cells, the three single PCS2 mutant clones replicated at significantly reduced levels. The 50% of tissue culture infectious dose (TCID_50_) of each viral stock was then determined as described in Methods. The TCID_50_ of all molecular clones with PCS2 mutations were 100–1000-fold lower than WT SIV or the PCS12 mutants (Figure 5B).

**Figure 6:**
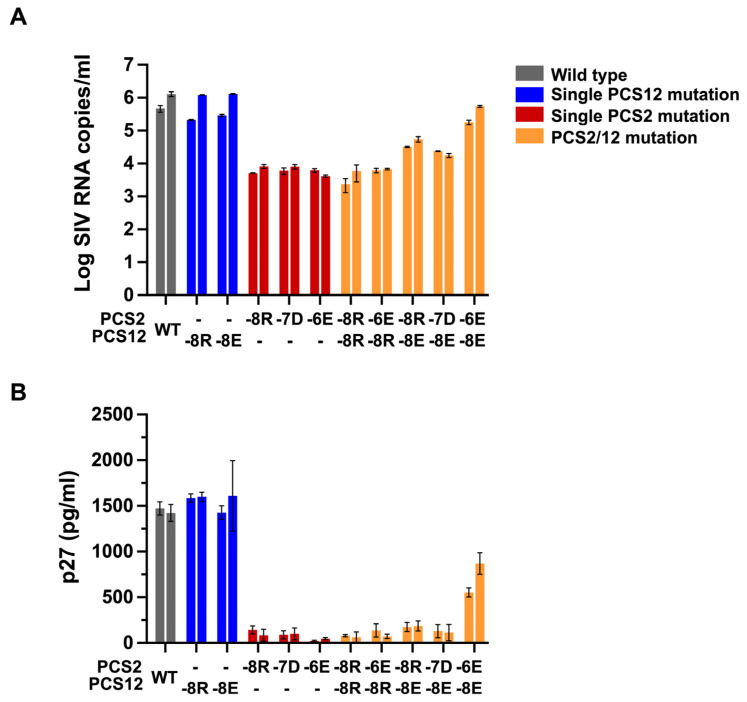
**Replication kinetics of PCS mutant virus in rhesus macaque PBMCs.** Viral replication of each PCS mutant virus was determined in primary rhesus PBMCs, isolated from two rhesus macaques with each PCS mutant variant and activated with ConA. Viral production was determined by real-time PCR for viral RNA (**A**) and ELISA for p27 (**B**) in culture supernatants harvested on day 7. Mean and standard deviation of two separate measurements are shown.

To confirm these observations, we used the same clones to infect PBMCs isolated from rhesus monkeys (Figure 6A,B). The three molecular clones with single PCS2 mutations were less competent compared to WT SIV, while the clones encoding single PCS12 replicated comparably to WT SIV. We found that the two molecular clones harboring double mutants, one in PCS2 (-6) and one in PCS12 (-8R or E), had WT SIV levels of RNA production in culture.

**Figure 7:**
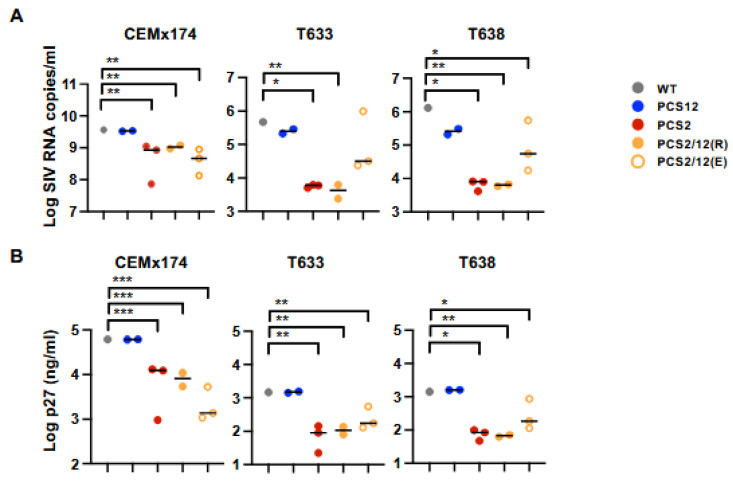
**Viral replication of PCS mutant virus in CEMx174 and rhesus PBMCs.** CEMx174 cells and rhesus macaque PBMCs (Sample ID: T633 and T638) were infected with viruses harboring a single amino acid mutation in either PCS2 or 12 site or combinations of two separate amino acid mutations in both sites. Infectivity of each virus relative to WT was evaluated. The comparison of viral RNA (**A**) and p27 (**B**) from the groups of virus mutants were analyzed using Kruskal–Wallis with Dunn’s multiple comparison test. The asterisks (*, ** and ***) indicate statistical significance (*p* < 0.05, *p* < 0.001 and *p* < 0.0001, respectively) between the indicated groups.

We next categorized all mutant virus permutations into groups based on the number of PCS mutations and performed comparison analysis for their replication kinetics (Figure 7A) and the production of mature virion as measured by p27-CA content (Figure 7B) between groups. A partial rescue of the PCS2(-6) mutant was noted by adding the PCS12 (-8E) mutation. This compensatory effect was increased in rhesus PBMCs as compared to CEMx174 cells. In sum, we demonstrate that the PCS2 mutation impairs viral replication in both primary rhesus PBMCs and the human T-B hybrid cell line CEMx174.

**Comparative fitness of PCS mutants during in vitro competition.** We then determined the relative fitness of a subset of PCS mutants compared to WT by competitive co-culture. Briefly, following established methods [26], two viruses (WT and mutant) were grown in the same cell culture; SIV RNA of mutant versus comparator in the culture supernatant was measured as our endpoint. Real-time PCR discrimination of the comparator virus by differential probe binding required the construction of a full-length infectious clone of SIV_mac239_ harboring two silent mutations in the *vif* gene (termed SIVmac239 Vif_mut_) that did not impact viral replication as described in Methods. All mutant stocks were assessed and titered by TCID_50_ and then evaluated in the two rhesus T cell lines (Rh444 and Rh445), as described in the Methods.

**Figure 8:**
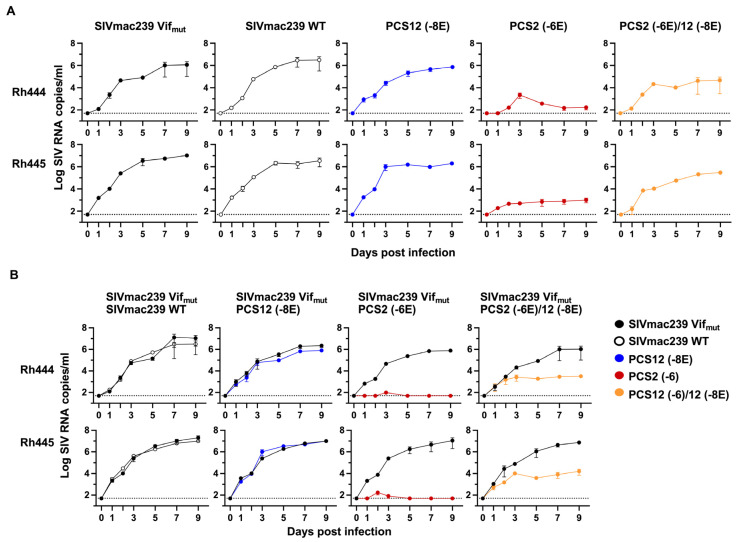
**Growth-competition dynamics between WT SIV and PCS mutant viruses in rhesus T cells.** A single virus growth (**A**) or competition assays with a reference virus (SIVmac239 Vif_mut_) and each PCS mutant virus (**B**) were performed in two rhesus T-cell lines using an MOI of 0.002. Representative plots indicate log_10_ SIV RNA copies/mL for both reference and comparator virus strains. Mean and standard deviation of two separate measurements are shown.

A comparison of the replication rate between SIVmac239 Vif_mut_ and the parental SIV WT demonstrated no significant difference in replicative fitness between them (Supplemental Figure S4). Next, each molecular clone harboring a PCS mutation was cultured and assessed independently compared to the SIVmac239 Vif_mut_ (Figure 8A). However, independent replication in separate culture vessels does not recapitulate the competition between strains in vivo. Therefore, the replication rate of the SIVmac239 Vif_mut_ to each PCS mutant clone was directly assessed by competition in the same culture vessel. We observed no differences in comparative replicative fitness between the SIVmac239 Vif_mut_ and PCS12 mutant. However, SIV Vif_mut_ outcompeted the PCS2 (-6) mutant and dominated the population. Only SIV Vif_mut_ was present, no PCS2-specific RNA was detected in culture supernatants 5 days after co-culture. The PCS2(-6)/12(-8E) mutant virus was detectable in cultures until day 9, although this virus never fully restored its replicative capacity to that of the parental WT SIV (Figure 8B).

## 4. Discussion

HIV-1 displays a remarkable degree of sequence diversity in its adaptation to natural-host or vaccine-elicited immunologic responses; this often results in viral escape to such responses. Not all escape mutations are advantageous, and many come at the expense of viral replication [28,29]. It has also been reported that initial escape variants, compromised in replication could eventually acquire compensatory mutations to restore viral replicative capacity [30]. Therefore, it is important to understand the viral fitness costs of viral escape from vaccine-induced responses, as this may directly impact HIV-1 vaccine design efforts.

Macaques vaccinated with PCS immunogens maintained higher CD4+ T cell counts than the controls. Moreover, vaccine-driven amino acid mutations around the 12 PCS sites were correlated with reduced in viral load after SIV challenge. The PCS are highly conserved among all major HIV-1 subtypes, making them strong candidates as universal vaccine targets [16,17]. Moreover, PCS sites require such high sequence conservation that even proximal alterations may be sufficient to interrupt viral protein processing, resulting in non-infectious progeny.

Here, we demonstrate that vaccine-driven mutations accumulated in the cleavage sites of Gag polyprotein precursor, specifically surrounding the PCS 2 region (CA/p2). These mutations were detrimental to viral replication due to defective protein cleavage and RNA incorporation. We show that the mutation near the spacer p2 motif disrupted Gag precursor processing, where p2 has been shown to be critical to the process of both simian and bovine immunodeficiency virus budding [31,32]. However, we also found that mutations occur in PCS12 region with high frequency, although they emerge later during SIV breakthrough relative to mutations in PCS2. Importantly, we found that the addition of a single PCS12 mutation resulting in amino acid substitution from a nonpolar, aliphatic glycine (G) to a negatively charged glutamate (E) partially rescued the PCS2 mutants’ infectivity and replication competence. Although the proteolytic cleavage of HIV/SIV Nef is correlated with its ability to stimulate virion infectivity [33,34,35,36], mutations in the PCS12 region did not completely rescue proteolytic processing. Thus, the compensatory mutations at PCS12 cannot fully compensate for the deleterious mutations at PCS2, implying that the highly conserved PCS is largely intolerant to mutation to preserve function, even at the cost of viral fitness.

In sum, we demonstrate that a vaccine-elicited immune response targeting protease cleavage regions can impair viral escape at specific sites adjacent to PCS regions, giving rise to breakthrough virus that is fitness-impaired. These findings suggest that vaccine-elicited immunity directed at immutable HIV-1 cleavage sites may constrain viral escape to variants of significantly impaired fitness that may be subject to facile immune clearance. These features highlight advantages of the PCS-targeted vaccine approach: (1) the reduced fitness of escape variants may lead to attenuated breakthrough infections, representing a clinical benefit when full protection is not achieved, and (2) the high conservation of PCS regions may lower the risk of natural resistance compared to previous vaccine strategies. Further preclinical and clinical studies are needed to test these hypotheses and assess the feasibility of translating this approach into a broadly effective HIV-1 vaccine.

## Data Availability

The original contributions presented in this study are included in the article/Supplementary Materials. Further inquiries can be directed to the corresponding author.

## Supplementary Materials

The following supporting information can be downloaded at https://www.mdpi.com/article/10.3390/v18030370/s1, Figure S1: Organization of SIV viral proteins and amino acid sequence alignment around the SIV cleavage site. Schematic representation of the protease cleavage sites at the Gag and Gag-Pol polyproteins and Nef protein of SIV (A). Amino acid sequence alignment of residues surrounding each cleavage site at −10/+10 regions among multiple SIV isolates (B). SIVmac239 was used as a reference. Residues that do not match with the SIVmac239 reference are highlighted in bold. GenBank accession numbers are shown in brackets: SIVmac239 (M33262), SIVmac251 (D01065), SIVmac142 (Y00277), SIVsmE660 (JQ86484), SIVsmE543 (U72748), and SIVsmPBj (L09212); Figure S2: Amino acid substitutions in 12 PCS regions identified in virus isolated from vaccine and control groups following SIVmac239 challenge. Numbers of amino acid substitutions per read including total (closed circles), conserved and non-conserved (open circles) within the −10/+10 amino acids flanking each of 12 PCS are shown. Symbol and error bars represent median and ranges in vaccinees (n = 11) and control (n = 5); Figure S3: Vaccine-derived PCS mutations and their frequency following SIVmac239 challenge. Prevalence and patterns of mutations in the CA/p2 cleavage site (PCS2) and the Nef cleavage site (PCS12). Four mutations, P → R change at position (-8), G → D change at position (-7), Q → E change at position (-6) of the PCS2 and G → R or E change at position (-8) of PCS12 are indicated. Y-axis: % of cynomolgus macaques with specified PCS mutation level (%CM with PCS mutation); Figure S4: Fitness comparison between the reference (Vifmut) virus and WT. Viral replication was determined in two rhesus T cell lines with the reference and WT SIVmac239 viruses. Log10 SIV RNA copies/mL were monitored viral production by real-time PCR for viral RNA. Mean and standard deviation of two separate measurements are shown; Table S1: Locations of the PCS point mutations and the production of replication competent PCS mutant viruses; Table S2: MID-tagged oligonucleotides used to generate amplicons spanning one or more protease cleavage sites in the SIVmac239 genome for each post-infection time point. The 10 bp Multiplex Identified (MID) Tag is highlighted in bold. The adaptor sequence and key sequence (TCAG) is shown 5′ to the MID tag. The virus-specific sequence is shown 3′ to the MID tag. Abbreviations: PC, protease cleavage site; 1–12, number represents a tar-geted protease cleavage site number; F, forward primer; R, reverse primer; MID, 10-base extended multiplex identifier set sequences.
