# Supplementary material for: Viral Escape from a Candidate HIV-1 Vaccine Targeting Protease Cleavage Sites Is Associated with a Dramatic Fitness Loss in SIVmac239-Infected Cynomolgus Macaques
Source: Viruses. 2026 Mar 17;18(3):370. doi: 10.3390/v18030370 (PMC13030808; doi:10.3390/v18030370)
Supplement: Supplementary file 1 [file viruses-18-00370-s001.zip › viruses-3706711-supplementary.pdf]

Supplemental Table S1. Locations of the PCS point mutations and the production of replication competent PCS mutant viruses.

**Site-directed PCS mutation**	**Amino acid sequence**			
	**PCS2 (CA(p27)/p2)**			**PCS12 (Nef)**
	**-8**	**-7**	**-6**	**-8**
SIVmac239 WT	P	G	Q	G
PCS12(-8)	P	G	Q	R
PCS12(-8)	P	G	Q	E
PCS2(-8)	R	G	Q	G
PCS2(-7)	P	D	Q	G
PCS2(-6)	P	G	E	G
PCS2(-8)/PCS12(-8R)	R	G	Q	R
PCS2(-7)/PCS12(-8R)	P	D	Q	R
PCS2(-6)/PCS12(-8R)	P	G	E	R
PCS2(-8)/PCS12(-8E)	R	G	Q	E
PCS2(-7)/PCS12(-8E)	P	D	Q	E
PCS2(-6)/PCS12(-8E)	P	G	E	E

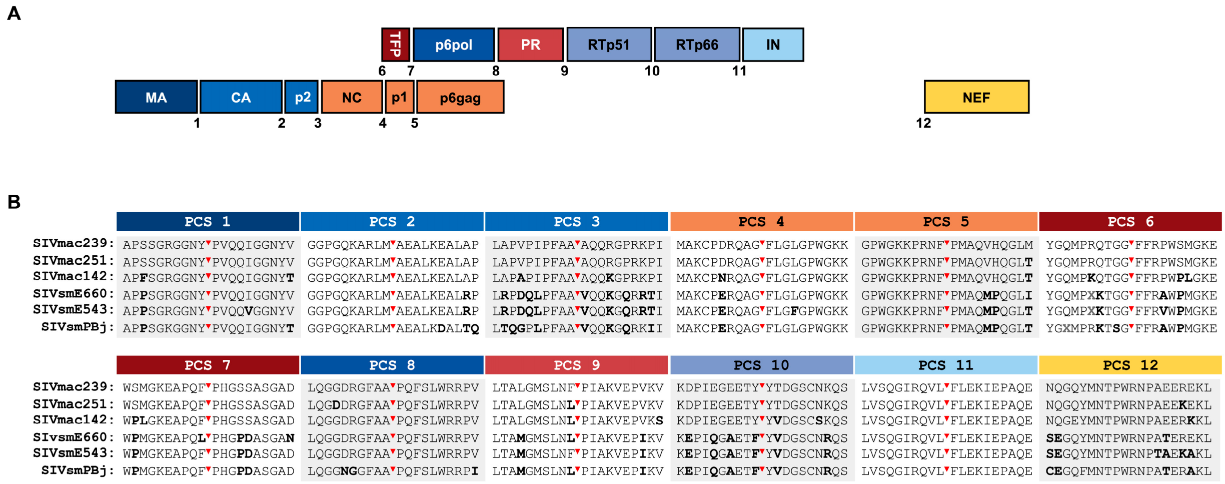

Supplemental Figure S1. Organization of SIV viral proteins and amino acid sequence alignment around the SIV cleavage site. Schematic representation of the protease cleavage sites at the Gag and Gag-Pol polyproteins and Nef protein of SIV (A). Amino acid sequence alignment of residues surrounding each cleavage site at -10/+10 regions among multiple SIV isolates (B). SIVmac239 was used as a reference. Residues that do not match with the SIVmac239 reference are highlighted in bold. GenBank accession numbers are shown in brackets: SIVmac239 (M33262), SIVmac251 (D01065), SIVmac142(Y00277), SIVsmE660 (JQ86484), SIVsmE543 (U72748), and SIVsmPBj (L09212).

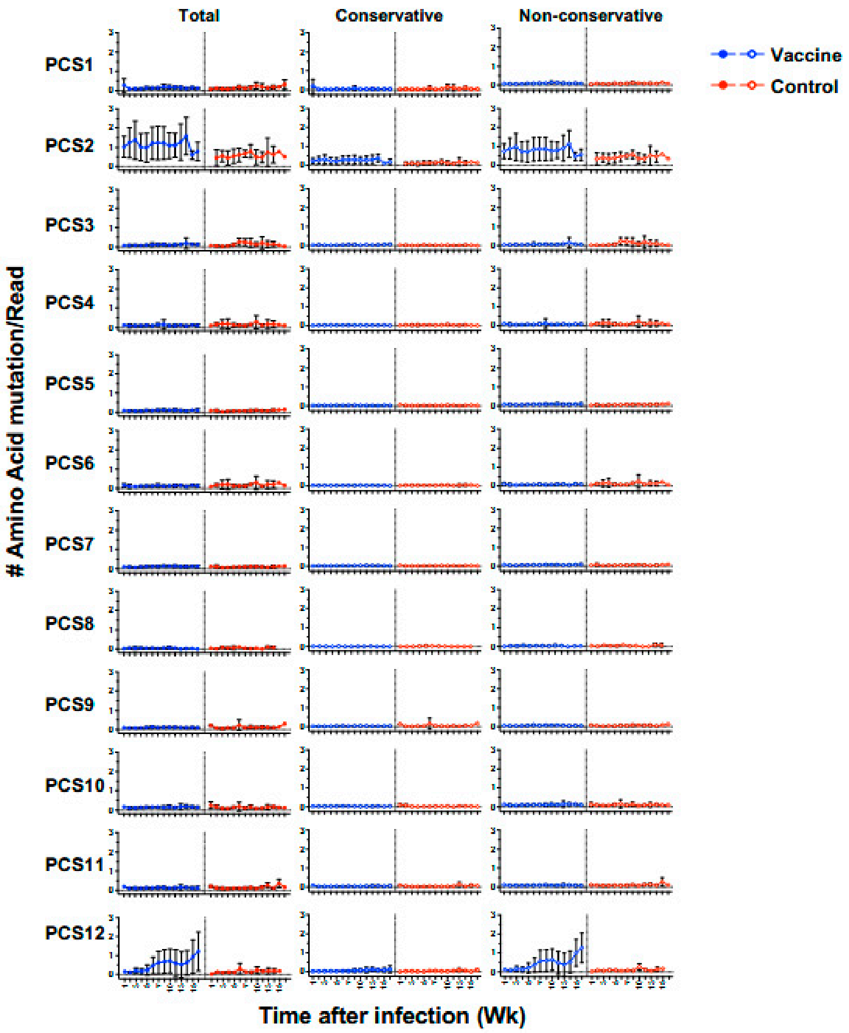

Supplemental Figure S2. Amino acid substitutions in 12 PCS regions identified in virus isolated from vaccine and control groups following SIVmac239 challenge. Numbers of amino acid substitutions per read including total (closed circles), conserved and non-conserved (open circles) within the -10/+10 amino acids flanking each of 12 PCS are shown. Symbol and error bars represent median and ranges in vaccinees (n=11) and control (n=5).

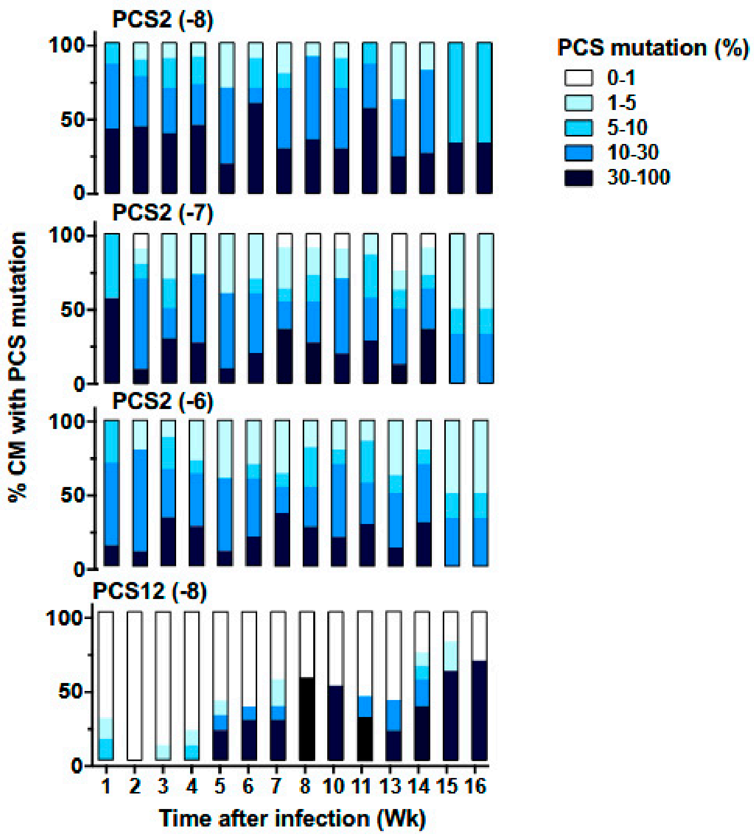

Supplemental Figure S3. Vaccine-derived PCS mutations and their frequency following SIVmac239 challenge. Prevalence and patterns of mutations in the CA/p2 cleavage site (PCS2) and the Nef cleavage site (PCS12). Four mutations, P→R change at position (-8), G→D change at position (-7), Q→E change at position (-6) of the PCS2 and G→R or E change at position (-8) of PCS12 are indicated. Y-axis: % of cynomolgus macaques with specified PCS mutation level (%CM with PCS mutation).

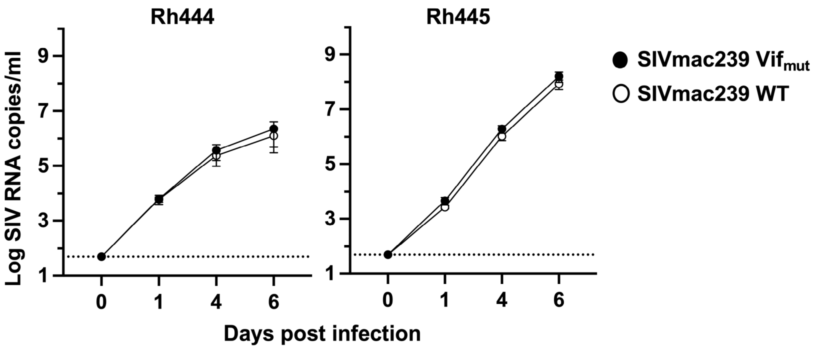

Supplemental Figure S4. Fitness comparison between the reference (Vif_{mut}) virus and WT. Viral replication was determined in two rhesus T cell lines with the reference and WT SIVmac239 viruses. Log_{10} SIV RNA copies/mL were monitored viral production by real-time PCR for viral RNA. Mean and standard deviation of two separate measurements are shown.
